# Clinical Application of Bioextracts in Supporting the Reproductive System of Animals and Humans: Potential and Limitations

**DOI:** 10.1155/2022/4766409

**Published:** 2022-03-27

**Authors:** Alicja Kowalczyk, Robert Kupczyński, Elżbieta Gałęska, Jose P. Araujo, Ewa Czerniawska-Piątkowska

**Affiliations:** ^1^Department of Environment, Animal Hygiene, and Welfare, Wrocław University of Environmental and Life Sciences, Chełmońskiego 38C, Wrocław, Poland; ^2^Mountain Research Centre (CIMO), Instituto Politécnico de Viana do Castelo, Rua D. Mendo Afonso, 147, Refóios do Lima, Ponte de Lima 4990-706, Portugal; ^3^Department of Ruminant Science, West Pomeranian University of Technology, ul. Klemensa Janickiego 29, Szczecin 71-270, Poland

## Abstract

There is an increasing demand of spices and herbs in developing countries due to the beneficial effects of plants and herbal preparations as medicines. The basic technological process of obtaining extracts from natural raw materials is extraction, consisting in etching with solvents. Plant extracts are extremely complex, multicomponent mixtures obtained from flowers, fruits, leaves, stems, twigs, or seeds of various plant materials. They are a rich source of polyphenols, flavonoids, phytosterols, carotenoids, and vitamins. The search for alternative methods of treatment is increasingly replacing the scientists' excessive focus on the healing properties of bioextracts. Recent research offers great hope for the development of alternative methods to improve the reproductive system. The use of animal models in experimental research has increased knowledge regarding the beneficial effects of bioextracts on both male and female reproductive systems and reproductive cells. Demonstrating the positive effect of plant extracts creates new opportunities for the use of biowaste, which is a by-product in various production sectors. The aim of this review is to present the functional properties of extracts of natural origin, a cross section of modern methods of their preparation, and a discussion of the possibilities of their use in the auxiliary reproductive system.

## 1. Introduction

Human infertility is described as the inability to conceive after 1 year of intercourse, and it is estimated that approximately 8–12% of couples worldwide are infertile [1]. Conception is influenced by many factors that affect both men and women. However, almost 40–50% of cases of failure in procreation are the result of male infertility. Chemotherapy, antibiotics, radiation, stress, pollution, and poor diet habits [2], environmental, occupational, and modifiable lifestyle factors influence fertility [3]. Male infertility can also be caused by genetic aberrations such as karyotyping abnormalities, Y-chromosome microdeletions, and mutations. Men with azoospermia and oligozoospermia have deletions on the long arm of the Y chromosome and mutations in the CFTR gene (cystic fibrosis transmembrane conductance regulator). Moreover, any mutations in the genes responsible for spermatogenesis and spermiogenesis, which are over 2,000, may lead to male infertility. The most common aberration in infertile men with nonobstructive azoospermia (MNOA) is Klinefelter's syndrome (KS; 47,XXY) [4].

In recent years, the use of spices and herbs has increased gradually in developing countries due to the useful effects of herbal preparations as medicines [5, 6]. Although many medicinal plants were proven to improve male fertility, unfortunately only a few of them have actually been used to treat it. In this regard, it is worth mentioning that two-thirds of the world's plant species have medicinal value; in particular, many medicinal plants have high antioxidant potential [7]. Recently, the growing literature on the effects of various plants on the reproduction of laboratory mammals has provided valuable information [8].

The search for alternative methods of treatment is increasingly replacing the scientists' excessive focus on the healing properties of bioextracts. The aim of this review is to present the functional properties of extracts of natural origin, a cross section of modern methods of their preparation, and a discussion of the possibilities of their use in the auxiliary reproductive system.

### 1.1. Bioextracts and Their Functional Properties

Plant extracts are concentrated extracts obtained from flowers, fruits, leaves, stems, twigs, or seeds of various plant materials. They are a rich source of polyphenols, flavonoids, phytosterols, carotenoids, and vitamins [9]. The chemical diversity of plants is very high, and plant-based food provides almost all the necessary nutrients for the human health, either directly or indirectly [10].

Medicinal products of plant origin are becoming an alternative to some drugs due to their low cost, easy availability, and lower side effects [11]. It is estimated that plant kingdom contains between 200,000 and 1,000,000 distinct secondary metabolites [12]. The combination of functional groups originates from this diverse and numerous set of metabolites. Plants are able to synthesize alcohols, aldehydes, alkyls, benzyl rings, steroids, and hydroxyls. These compounds often interact with each other, causing beneficial effects for the plants that synthesize them. The combination of polyphenols, carotenoids, or glucosinolates causes anti-inflammatory, antioxidant, and antimicrobial effects. The bioactive effect of plants is mainly due to the presence of alkaloids, saponins, terpenoids, and polyphenols [13]. Alkaloids are natural nitrogen-containing compounds commonly found in land plants with anesthetic (cocaine), stimulant (caffeine, nicotine), analgesic (morphine), antibacterial (berberine), anticancer (vinblastine), antispasmodic (atropine), and antiasthma properties (ephedrine) [14]. Saponins are compounds related to the color of plants found in leaves, stems, roots, and flowers. They can induce humoral and cellular immune responses. When used as vaccine adjuvants, they are associated with a broad secretion of cytokines [15]. As secondary metabolites, terpenoids show a wide range of activities, such as hepatoprotective, hypoglycemic, immunomodulating, anti-inflammatory, antioxidant, and antitumor activities. For instance, lupane, oleanane, and ursanes are pharmacologically active without any toxic effect [16]. Polyphenols are mainly found in vegetables (broccoli), fruits (grapes, blueberries), legumes (soybeans), grains, plant-based drinks, and chocolate. They are divided into flavonoids (flavones, flavonols, flavanones, flavanols, isoflavones, and anthocyanins), phenolic acids (e.g., caffeic acid), stilbenes (e.g., resveratrol), tannins, and lignans (e.g., linseed) [17]. Polyphenols have an effect on heart diseases, inhibit establishment of atherosclerotic plaques, and regulate lipid metabolism. Flavonoids as a group of polyphenols are widely used as antioxidants and anti-inflammatory agents [18].

#### 1.1.1. Water-Glycerin Extracts

Water-glycerin extracts are based on a mixture of water and glycerin in various proportions. Glycerin has strong moisturizing and moisture-retaining properties in the skin. Moreover, it facilitates the penetration of active ingredients into the skin and, as a cryoprotectant, it protects cells against the negative effects of cryopreservation [19].

#### 1.1.2. Hydroalcoholic Extracts

Hydroalcoholic extracts are based on a mixture of water and alcohol in various proportions. Alcohol acts as both a solvent and a preservative. It has been shown that aqueous and methanolic extracts from wild carrot canopy are anti-inflammatory, antiulcer [20], and anticancer [21].

#### 1.1.3. Oil Extracts

In this type of extract, it is possible to etch substances of lipophilic nature. A study by Shebaby et al. [22] showed that oil extracts may possess both antioxidant properties and promising anticancer properties.

#### 1.1.4. CO_2_ Extracts

Obtaining extracts using supercritical carbon dioxide is an increasingly popular and ecological method of producing CO_2_ extracts. In the supercritical extraction process, the supercritical fluid is the solvent. Supercritical condition means that the pressure and the temperature conditions have exceeded the critical point (varies according to the substance); therefore, the physical properties of the substance have changed. Under supercritical conditions, the physicochemical parameters of the fluid are in an intermediate position between the liquid and gaseous states. CO_2_ extracts are characterized by low viscosity (similar to gases) and relatively high density (similar to liquids). Due to these characteristics, supercritical fluids have good penetration and transport properties, making them a useful solvent for the extraction process [23].

Supercritical CO_2_ is a physiologically inert and nontoxic gas, which does not react with most substances of natural origin. It is nonflammable, cheap, and widely available and, most importantly, possesses bacteriostatic properties. Carbon dioxide reaches a supercritical state at temperatures just above 31°C. Some advantages of this method of extraction from CO_2_ are the following: no residual solvents; low process temperature; the process occurs without air access, which protects biologically active compounds against oxidation; and it enables the regulation of the solubility of individual compounds by changes in pressure and temperature of the process [24].

### 1.2. Methods to Obtain Natural Extracts

The basic technological process to obtain extracts from natural raw materials is extraction, consisting in etching (based on the diffusion phenomenon) with solvents.

Two techniques distinguish it from the method of solvent administration: periodic extraction methods, in which the solvent is fed once or in parts, and the continuous extraction method, in which the raw material is in constant contact with the fresh solvent [25].

Extraction is a process of separating components from mixtures of solids and liquids, in which a component from the extracted phase passes into a solvent under the influence of a concentration difference. In other words, extraction consists in extracting active ingredients from purified, dried, and shredded plants with a suitable solvent: water, ethyl alcohol, glycol, glycerin, or isopropyl myristate.

Advances in sample preparation technology hold significantly better results than conventional methods, especially by reducing the consumption of organic solvents and minimizing sample degradation. Thanks to new technologies, it is possible to eliminate undesirable and insoluble components from the extract. These modern methods are mainly microwave-assisted extraction (MAE), ultrasound-assisted extraction (UAE), supercritical fluid extraction (SFE), solid phase microextraction (SPME), and Soxhlet wave. The last method is combining the Soxhlet method with microwave. We found two main advantages in this method: the fast heating power of microwaves and the simplicity of the Soxhlet. The researcher has the ability to recover the solvent, which is not the case with ordinary MAE. However, this method is not yet widely used.

The prevailing classical methods are quite simple and standardized. Notwithstanding, they may be insufficient and slow, resort to large amounts of organic solvents, and destroy thermally labile components. The use of conventional methods entails quality problems. Particular problems are visible in inconsistencies, security, and performance. Modern processes brought the possibility to eliminate additional steps of sample purification and concentration prior to chromatographic analysis. The result is improved extraction efficiency and selectivity [26]. Such changes aim to obtain the therapeutically desired portion and to eliminate the inert material. This goal can be achieved with selective solvents and methods. More attention has been drawn to herbal remedies and natural health products around the world. In the production of herbs, every effort is made to use the most appropriate extraction technologies in order to produce extracts of a certain quality, while obtaining the smallest possible differences between batches. Nonetheless, this treatment may increase the scale of the extraction. Maintaining appropriate standards for the procedures of the extraction process greatly affects the quality of the product obtained. If we wish to understand the bioactivity of raw extracts, the optimization of the extraction methods is key for obtaining the widest possible range of phytochemicals. The choice of the method to isolate active ingredients from natural sources in order to obtain the best efficiency and the highest purity depends primarily on the type of compounds and the selected raw material [27].

#### 1.2.1. Microwave-Assisted Extraction (MAE)

The electromagnetic energy of microwaves, absorbed by the material, is interchangeable with thermal energy. 2450 MHz (2.45 GHz) is the most used frequency in commercial applications of microwaves with an output power of 600–700 W [28]. MAE is a simple, economic, and environmentally friendly technique utilized to extract biologically active compounds from the chosen plant materials [29]. Contrary to the classical methods of conductive heating, these microwaves heat the entire sample at the same time. The advantage of such heating in the case of extraction is that it breaks weak hydrogen bonds. These bonds favor the rotation of the molecules' dipoles [30].

Increasing the temperature causes the moisture to evaporate and creates a high vapor pressure. The application of these factors breaks the cell wall of the substrate, thus releasing the contents into the solvent. Most MAE operations use solvents that hold a high dielectric constant and exhibit the ability to strongly absorb microwave energy. The selectivity of extraction and the possibility of interaction with the medium can be corrected using microwaves and solvent mixtures.

MAE can be used in two variants: one works in a closed vessel, *i.e.*, controlled (and increased) pressure and temperature; and the other works in an open vessel under atmospheric pressure. The first technology is called the microwave-assisted pressure extraction (PMAE) method, and the second is the microwave-assisted concentrated extraction (FMAE) [31].

In a closed vessel system, the temperature of the solvent can be raised above its atmospheric boiling point. This process will increase both the extraction's rate and efficiency [30]. The temperature in the vessels rises by applying suitable pressure. The closed vessel creates optimal conditions for volatile compounds, while the maximum temperature in open systems is obtained on the basis of the boiling point of the solvent employed [32]. Compared with closed systems, open systems ensure greater safety of sample handling during extraction, as well as greater efficiency of the overall process [30].

#### 1.2.2. Ultrasonication-Assisted Extraction (UAE)

Ultrasonication-assisted extraction relies on high intensity and frequency sound waves and their interaction with materials. The UAE is a potentially useful technology as it does not require complex instruments and is relatively cheap. The process can be carried out both on small and large scales [33]. UAE is the ultrasonic effect of acoustic cavitation. Under the influence of ultrasound, solid and liquid particles vibrate and accelerate. During the reaction, the solute quickly diffuses from the solid phase to the solvent [34].

The applied extraction by means of ultrasound was not only more efficient; this method is also convenient to recover and purify the active ingredients used. Sonication-assisted extraction may be beneficial for thermally labile compounds because it can be performed at lower temperatures [35]. Thanks to this application, the technique is very useful for the isolation and purification of bioactive components [36]. This procedure also has a disadvantage: the occasional (but known) detrimental effect of ultrasonic energy (>20 kHz) on active ingredients in medicinal plants. The harmful effect occurs through the formation of free radicals. As a consequence, undesirable changes in drug molecules occur [37].

#### 1.2.3. Supercritical Fluid Extraction (SFE)

SFE is an old solvent extraction technique. Its commercial application was slow as it was done with sophisticated and expensive high-pressure equipment [38]. SFE is an extraction and separation method, whose design and operational criteria are well known and, therefore, well established [39]. The transport properties of the fluids are advantageous because the oscillation near their critical points allows deeper penetration of the solid plant matrix. This makes this method more efficient and faster than the conventional extraction method using organic solvents. Extraction occurs in a high-pressure device in a batch or continuous manner. In both cases, the supercritical solvent encounters the material, thereby separating the desired product. To prepare the samples, cylindrical extraction vessels are used [37]. During batch processing, the solid is placed in the extraction vessel. The supercritical solvent is fed until the target extraction conditions are achieved. This technology is now recognized as an effective technique for performance analysis compared with other existing chemical analysis methods. SFE is employed in the qualitative and quantitative identification of natural products' ingredients. It can also be used for thermolabile compounds [40].

## 2. The Importance of Bioextracts in the Males' Reproductive System

Fertility is closely related to the male's ability to produce sperm capable of fertilizing oocytes and conceiving new life. Fertility is influenced by many factors, including age, nutrition, genetics, and health status. Nevertheless, good semen quality is required to achieve adequate fertility in mammals [41].

### 2.1. Nutritional Additives

Various natural products present positive and negative effects on male fertility (Table 1).

A recent study by Zare et al. [42] found that date palm (*Phoenix dactylifera* L.) fruit extract (DPFE) contains a high concentration of natural antioxidants. DPFE consumption before formaldehyde (FA) administration could partially ameliorate the reduced testosterone, sperm, and testicular parameters due to FA. Afrigan et al. [43] found that a *Matricaria chamomilla* extract at a dose of 500 mg/kg reduces the adverse effect of formaldehyde on the reproductive system of male rats. Formaldehyde increases the rate of apoptosis of reproductive cells and reduces sperm parameters. Moreover, the flavonoids contained in the test's extract contribute to an increase in the content of luteinizing hormone and testosterone. The antioxidants contained in the extract also protect Leydig cells from free radicals that interfere with steroidogenesis [43]. Furthermore, the authors showed that the sperm count, motility, and viability were significantly higher in the test groups *vis-à-vis* the control group. Other researchers have proven that the addition of *F. parviflora* alcoholic extract to the nutritional dose has a positive effect on the male reproductive system of rats, stimulating a rise in the number of sex cells (spermatogonium, spermatocytes, sperm, and Leydig cells) [75]. In mice, oral administration of the hydroalcoholic extract of *Nasturtium officinale* L. reduced the effects of damage to the tests caused by oxidative stress and improved the biological parameters of sperm in terms of sperm motility and viability [76]. El-Desoky et al. [77] proved the beneficial effects of *Moringa oleifera* leaf extract. Supplementation of the extract with 50, 100, and 150 mg/kg body weight significantly increased the concentration of rabbit sperm exposed to heat stress and had a positive effect on the motility of progressive sperm cells, normal sperm morphology, the degree of intact acrosomes, and the number of sperm characterized by maintaining the continuity of the cytoplasmic membrane. Other studies [78] have provided evidence that *Aspalathus linearis* extract can increase sperm velocity, can protect the acrosome structure, and tends to preserve membrane integrity up to 96 hours of liquid boar semen storage. Additional evidence for the beneficial effects of the extracts was also provided by Hemmati et al. [79], who showed that roosters receiving 2000 mg/L of knotgrass extract had a significantly higher percentage of motile and normal sperm than the control group. Moreover, the use of the same dose of the aforementioned extract significantly reduced the abnormalities in the sperm head, midsection, and tail. The researchers observed the highest number of live sperm with the highest motility and normal morphology in the group receiving 2000 mg/L of knotweed extract. Other authors have proved that Gilaburu (*Viburnum opulus L.*) fruit extract (100 mg/kg) alleviates testis and sperm damage induced by taxane-based chemotherapeutics [80]. According to Peiris et al. [81], aqueous leaf extract (ALE) from *Cardiospermum halicacabum* has the ability to enhance the fertility of male Wistar rats. The research was conducted for 30 days with 100 mg/kg and 200 mg/kg body weight ALE. A greater number of sperm was obtained in the epididymides of rats, which were characterized by greater motility and a higher level of testosterone in the plasma [81]. The authors indicate that the increase in sperm motility is the result of glucose metabolism, as a result of which pyruvate is formed. Moreover, the tested extract of *C. halicacabum* shows antioxidant activity due to the content of flavonoids, which protect sperm against oxidative stress and reactive oxygen species (ROS) [81, 82].

### 2.2. Spermatozoa Preservation

The preservation and processing of semen is a risk factor for sperm viability and motility, potentially leading to a reduction in fertilization efficiency. A number of extracts obtained from natural sources may have a protective or antioxidant effect on sperm cells, thus increasing the usefulness of using insemination doses in artificial insemination. Low fertility is largely due to a reduction in sperm fertilization during the cryopreservation process. In fact, about 50% of the sperm in the ejaculate survive the current freeze-thaw process [83, 84].

During the preparation of semen for cryopreservation, semen plasma containing antioxidants is removed. Therefore, during storage, semen is exposed to oxidative stress, and, as a result, the sperm, including its DNA, is damaged. According to Sengupta et al. [85], the grape pomace extract neutralizes reactive oxygen species due to its rich content of polyphenols. This was demonstrated in a study on bovine sperm: the extract increased sperm motility and viability.

Research reports that *Chlamydomonas* extract is able to delay the damage caused to sperm stored *in vitro* in the environment. The CASA analysis showed that supplementation with *Chlamydomonas* extract was able to prevent a rapid decline in sperm motility, especially at concentrations from 1 to 5 *µ*g/ml (with respect to 6, 12, and 24 hours). At the same time, extract concentrations ranging from 5 to 10 *µ*g/ml led to a significant maintenance of cell viability [86]. Asadmobini et al. [87] investigated the effect of *Tribulus terrestris* extract on the motility and viability of human sperm after cryopreservation. The study showed that the protective effect of *Tribulus terrestris* (40 and 50 *μ*g added to semen)—improving sperm motility and viability—may result from its antioxidant properties. Based on such results, the researchers concluded that *Tribulus terrestris* could be used as a safe therapeutic alternative to the current methods of treating motility dysfunction in men. On the other hand, a new study by Naderi et al. [88] proved the beneficial effect of *Ferulago angulata* extract on the biological parameters of cryopreserved buck sperm. The results showed that *Ferulago angulata* extract added to the semen (0.002%, w/v) improves sperm cryopreservation efficiency, viability, membrane integrity, and fertilization capacity and also reduces ROS production. Other authors focused on investigating the parameters of *in vitro* fertilization by adding a tannin-rich plant extract (a mixture of horse chestnut and Quebracho wood extracts (60/40 v/w)) to frozen thawed boar sperm [89]. Although they did not show a positive effect of this extract on the motility or viability of boar sperm, the oocytes inseminated with thawed sperm pretreated with all different concentrations (5, 10, 20 *µ*g/ml) of the extract showed a significant increase in the penetration coefficient compared with the control group. Additionally, 5 *µ*g/ml of the above extract had a positive effect on the overall efficiency of fertilization. These results encourage the use of this supplement in thawing medium as post-thaw fertility is a limitation for the large-scale use of frozen semen [90–93].

### 2.3. Potential Mechanism of Action of Active Compounds

According to Adewoyin et al. [82], sperm oxidative stress and inflammation in response to infections or tissue damage are related. With male infertility, high levels of ROS in semen often coincide with high levels of proinflammatory cytokines in the semen. Inflammation inhibits spermatogenesis and sperm maturation processes. Moreover, sperm motility is limited by an increase in the stiffness of the sperm flagellum membrane [82]. Mammalian sperm has a cell membrane rich in unsaturated fatty acids; therefore, these cells are highly sensitive to oxidative stress [94].

Antioxidants are a group of compounds that either neutralize the action of ROS or stop its production. The most important representatives of the group are vitamin A, tocopherol, tocotrienols (vitamin E), vitamin C, and beta-carotene. It has been shown that selenium, zinc, carnitine, arginine, and vitamin B-12 have the ability to increase the number of sperm and to positively affect its motility [82]. During the treatment of male infertility, the beneficial effect of antioxidants such as vitamin C, coenzyme Q10, vitamin E, and glutathione on male reproduction function was observed. Endogenous and exogenous antioxidants are important for maintaining the redox balance. The endogenous ones include superoxide dismutase (SOD), catalase (CAT), glutathione peroxidase (GPx), and the exogenous ones are vitamin C, E, and polyphenols [82].

Mammalian sperm plasma (SP) is a rich secretion of the testes, epididymides, accessory sex glands, and antioxidant, nonenzymatic, and enzymatic components that influence the balance of ROS. Nonenzymatic ingredients are, for example, albumin, taurine, and melatonin. In turn, the enzymes with antioxidant activity are superoxide dismutase (SOD), catalase (CAT), glutathione peroxidase (GPx), and glutathione reductase (GSR). CAT, SOD, GPx, and GSR are the main antioxidants found in mammalian semen. Each of these enzymes participates in a specific reaction: SOD catalyzes the dismutation of the superoxide anion to oxygen and hydrogen peroxide; CAT catabolizes hydrogen peroxide to water and oxygen; GPx oxidized reduced glutathione; and GSR reduces oxidized glutathione. The joint action of these enzymes is essential for adequate antioxidant capacity in mammalian semen [94]. The composition of the semen plasma varies between species and individuals. Moreover, higher SOD activity was noted more in donkey semen than in humans', rabbits', pigs', cattle, horses', or cocks' [94].

The effect of medicinal plants and their bioextracts on the improvement of male reproductive functions is most often associated with their antioxidant activity. These substances have a positive effect on spermatogenesis and steroidogenesis [82]. In order to select the suitable supplementation and determine the adequate doses of antioxidants contained in bioextracts, the level of oxidative stress should be measured. According to Adewoyin et al. [82], infertility treatment and antioxidant supplementation aimed at improving fertility are still in the initial stage of research. The use of plant extracts to improve reproductive function is promising, but clinical trials are needed to determine the suitable substance and its dose.

## 3. The Importance of Bioextracts in Females' Reproductive System

Medicinal plants and natural products are widely used to increase or regulate female fertility (Table 2). These included the passive effects of medicinal plants on female fertility: stimulation of a normal pituitary gonadotropin-releasing hormone (GnRH) response; improvement of normal pulsatile FSH and LH secretion; ovulation induction; increased secretion of steroid hormones in ovarian cells; having estrogen and progesterone effects; and directly regulating ovarian function, at least in part, by inducing cytokine secretion [95, 96].

The effectiveness of the hydroalcoholic extract of *Anthemis nobilis* was studied in polycystic ovary syndrome induced in rats with a single dose of estradiol valerate. Histological studies showed that the animals dosed with 50 mg/day showed small cysts and less inflammation, with a decrease in serum levels of the hormone estrogen. In turn, several studies reported that *Calendula officinalis* flower extracts exerted estrogenic activity in ovariectomized animals [97, 98]. Tavana et al. [99] examined the effect of different concentrations of the aqueous saffron extract (*Crocus sativus*) (SAE) on the *in vitro* maturation process of immature mouse oocytes. They showed that the maturation rate was significantly higher in all groups treated with various concentrations of SAE compared with the control group. Moreover, concentrations of SAE 10 and 5 *μ*g/ml in the medium increased the oocyte fertilization index and *in vitro* development capacity compared with the control group. This suggests that adding adequate amounts of SAE to the maturation medium can improve oocyte maturation and embryo development. Other studies found that SAE was effective in relieving the symptoms of premenstrual syndrome. They point out to a significant difference in SAE efficacy in terms of total daily premenstrual symptoms [100, 101]. Interestingly, it has been shown that the crude aqueous extract of *J. repens* (except the root) can act on uterine contraction *in vitro*. The results showed that the crude aqueous extract of *Jussiaea repens* at a dose of 40 mg dry extract/30 ml physiological fluid in a bath on an isolated nonpregnant uterus of female rats *in vitro* caused a significant increase in force and frequency of contractions than normal. The results (as a percentage) were compared with the effect of oxytocin in the presence of atropine. The extract has been shown to act as oxytocin, which has been antagonized by atropine [102, 103]. Yavangi et al. [104] found that *F. vulgare* seed extract administered orally was effective in improving the determinants of conception in women and could be used as a complementary therapy. The use of *F. vulgare* positively improved the quality of oocytes and fertility indices in women.

## 4. Natural Products to Regulate Libido

It has been found that libido (sex drive) can be modulated by various factors including diet, seasons, age, environment, and genetics [105]. Low libido is categorized as male sexual dysfunction, caused by low testosterone levels [106]. The sex drive can be increased with the help of aphrodisiacs, for example, herbal and plant products. These substances increase sexual performance or support the proper functioning of the sexual organs [105]. Aphrodisiacs can increase erection, blood flow, or relax smooth muscles through hormonal changes [105]. It has been found that the administration of plant extracts may affect fertility by improving sperm parameters, reducing ovarian dysfunction, or increasing hormones such as progesterone or testosterone. In addition, many herbs and plants are commonly rich in antioxidants that protect the reproductive system against oxidative stress that can damage sperm and oocytes [107].

Herbal extracts from *Tribulus terrestris* L. (TT) are commonly used in China, Korea, and Africa for ailments related to hypertension, rheumatism, or impotence [46]. The positive effect of TT extracts on libido or erectile dysfunction is due to the presence of steroid saponins, phytosterols, phenolic compounds, tannins, terpenoids, amino acids, and proteins; the most important of which is protodioscin (PTN) found in *Trigonella* and *Dioscorea* species [46, 105, 108]. In studies with TT-treated rats, an increase in testosterone content was shown, which indicates that this extract influences androgen metabolism [46]. In turn, in other studies by Bharavi et al. [108], the protective effect of TT extract against cadmium-induced testicular damage was found in rats. The protective potential of TT is due to its antioxidant and metal chelating properties as well as possibly the increased testosterone production in Leydig cells [108]. Similar antioxidant properties of *Tribulus terrestris* L. extract were demonstrated in relation to damage to the male reproductive system by cyclophosphamide (CP) in mice. CP is commonly used in the treatment of leukemia and lymphoma, among others, and men with these diseases treated with CP were characterized by azoospermia and oligospermia. During the research, animals were treated with *Tribulus terrestris* dry extract diluted in distilled water, orally administered, and daily for 14 days at a dose of 11 mg/kg body weight, and CP (100 mg/kg bw) was administered once intraperitoneally, 1 hour after the last administration of the dry extract *Tribulus terrestris* (14 days). The studies showed the protective potential of TT extract on the male reproductive system of mice against damage caused by CP, the effects of which are associated with the occurrence of oxidative stress [46].

Another extract that has a positive influence on libido is the extract from the *Lepidium meyenii* Walp. plant [109]. It is mainly grown in South America and is characterized by improved male and female reproductive performance [109–111]. This extract has a modulating effect on menopausal symptoms [112]. It has been proven to be spermatogenic, increase fertility, and improve sperm parameters [110, 112]. Maca (*Lepidium meyenii* Walp.) extract has been suggested to act as an aphrodisiac through its lipid content, which improves sexual function and sperm mobility [110]. Drying has been shown to be a crucial process for the extraction and formation of maca bioactive metabolites [112]. In research conducted by Ohta et al. [111], it has been proven that the hydroalcoholic extract of *Lepidium meyenii* Walp. administered for 6 weeks to rats increases plasma testosterone levels and increases the weight of the reproductive organs. A greater weight of seminal vesicles was also shown in rats given a hydroalcoholic extract compared with controls [111].

The bioactive substance obtained from turmeric rhizomes is curcumin, which has been widely used in Chinese medicine. Curcumin exhibits antibacterial, anti-inflammatory, and antioxidant activities [113, 114]. In the male reproductive system, it acts as a testicular protective factor against oxidative stress that is induced, for example, from exposure to toxic metals such as cadmium. Curcumin has been shown to increase levels of oxidoreductases, like superoxide dismutase [113]. In studies on mice, which were injected intraperitoneally with CdCl_2_ in a dose of 2 mg/kg body weight and curcumin (50 mg/kg body weight) for 10 days, a decrease in sperm motility and a reduction in testosterone content as a result of administration of cadmium solution were proven. In turn, because of curcumin treatment, the mice sperm quality was better, and the testosterone content and the antioxidant capacity increased. Also, a lower rate of sperm deformity in the epididymides was obtained in the group of mice treated with curcumin compared with the group that was administered cadmium alone. It has also been proven that curcumin increases the level of glutathione peroxidase, or superoxide dismutase, and reduces the content of hydrogen peroxide [113].

Another medicinal plant used in the treatment of many diseases is *Senecio biafrae*, the leaves of which are rich in secondary metabolites, such as terpenoids or sesquiterpenes [115]. It has been proven that the ethanol extract of *Senecio biafrae* influences the maturation of oocytes in immature female rats, as well as improving fertility indicators [107]. In the research, animals were administered at doses of 8, 32, and 64 mg/kg of aqueous extract, respectively, for 20 days. There was an increase in the hormonal activity of estrogen, progesterone, luteinizing, and follicle-stimulating hormones [115].

The Southeast Asian plant *Eurycoma longifolia* (EL) has been found to be a natural herbal remedy for male sexual dysfunction, including improving libido by increasing blood testosterone levels [82, 106].

The effects of medicinal plants on male fertility, including libido, are mainly associated with antioxidant properties. According to Thu et al. [106], the action of this type of plants improves the process of spermatogenesis and steroidogenesis and may also have a protective effect against oxidative stress.

## 5. Side Effects of Using Extracts

The use of herbs and plants in the form of extracts, due to the rich composition of antioxidants or flavonoids, may also have adverse effects [107]. Various types of supplements containing herbal and plant extracts can cause intense side effects [105, 116]. The most common side effects of natural products to increase libido are nausea, vomiting, diarrhea, and dizziness [105, 116]. The main natural products that may have a negative effect are, among others, *Tribulus terrestris* and yohimbine [105].

TT extract has been shown to cause side effects at a dose of 7.5 ml of 60% solution for 4 weeks twice daily in women, such as abdominal pain, nausea, vomiting, and diarrhea [117]. Toxicity to the nervous system is also documented at a dose of 20 mg/kg in rats. At that time, abnormalities of astrocytes, a reduction in neuron uptake, and a reduction in the stimulation of the hypothalamic nucleus were detected [105]. The side effects of *T. terrestris* L. extract may concern allergic reactions or effects on the gastric mucosa [118].

It has been proven that yohimbine, an indole alkaloid obtained from the bark and leaves of the yohimbe tree (*Pausinystalia johimbe*), has a hallucinogenic effect, causes an increase in blood pressure, accelerated heart rate, bronchospasm, and shortness of breath. A dose in the range of 200–5000 mg of yohimbine is associated with neurotoxicity or nephrotoxicity [105].

There are situations in which substances and products intended to increase sex drive have a negative effect on the body, causing side effects. When using natural extracts, the correct dosage is important [105].

## 6. Conclusion

Recent years of research give great hope for the development of alternative methods to support the functioning of the reproductive system. Experiments in animal models provide evidence of the beneficial effects of bioextracts on both the male and female reproductive systems and reproductive cells. Establishing the positive effect of plant extracts creates new opportunities for the use of biowaste, which is a by-product in various production sectors [119]. However, further investigation is needed to evaluate the correct mechanism on testicular and sperm parameters, as well as the selection of appropriate doses. Moreover, the development of an appropriate dose for a specific extract may be difficult due to the variable chemical composition of individual plant species and the different types of extracts produced from them and the extraction method.

## Figures and Tables

**Table 1:** Summary of research work of importance of natural products for male fertility.

	Product	Effect	Reference
Positive	Date palm (*Phoenix dactylifera* L.) fruit extract (DPFE)	DPFE can be applied as an effective protecting agent to reduce the deleterious effects of formaldehyde	[42]
	*Matricaria chamomilla* extract	Reduces the adverse effect of formaldehyde on the reproductive system of male rats. The antioxidants also protect Leydig cells from free radicals that interfere with steroidogenesis	[43]
	*Tribulus terrestris* Linn.	Improvement of sperm motility and efficiency	[44–46]
	*Cistanche tubulosa*, echinacoside, and water methanol extract *Loranthus micranthus*	Effect on the increase in testosterone levels and the enzymes CYP11A11, CYP17A1, 3*β*-HSD, 17*β*-HSD	[47, 48]
	Acacia hydaspica ethyl acetate extract, mature garlic extract, diallyl sulfide, water methanol extract *Loranthus micranthus*, methanol extract of *Carissa opaca* leaves, *Teucrium polium* extract, and *Thymus algeriensis* extract	Reduction of oxidative stress, reduction of free radicals (NO, TBRAS), increase of oxidative enzymes (SOD, CAT, GSH, GST, and GPx), and reversal of infertility caused by oxidative stress	[47, 49–54]
	*Echinacea purpurea* ethanol extract, *Lycium barbarum* polysaccharide, and white tea	Restoration of reproductive function and blood glucose levels in mice with STZ-induced diabetes	[55–57]
	Gosha-Jink-Gan	Potentially restores spermatogenesis	[58, 59]
	Saikokaryukotsuboreito	Improves spermatogenesis and testosterone levels	[60, 61]
	Wuzi Yanzong	Reduces oligoasthenozoospermia by regulating sex hormones	[62]
	*Apis mellifera*	Antioxidant, antihyperglycemic, and antidiabetic	[63,64]
	Barley, royal jelly	Decrease in free radicals and increase in antioxidant enzymes	[65,66]
	Cinnamon, *Ficus asperifolia* water extract	Reduction of oxidative stress and control of lipid components, improving fertility	[67, 68]

Negative	*Olea europaea* water-alcohol extract and permethrin	Decline in reproductive function even at doses as low as 50 and 35 mg/kg, respectively	[69, 70]
	*Tripterygium* glycoside extract	Inhibition of the activity of hormone receptors critical in the fertilization process	[71]
	The dose of *Ricinus communis* L. water extract was higher than 100 mg/ml, and the dose of ethanol extract from *Spondias mombin* L. and red cultivar *Allium cepa* methanol extract exceeded 100 mg/kg	Adverse fertility	[72–74]

**Table 2:** Summary of research work of importance of natural products for female fertility.

	Product	Effect	Reference
Positive	*Apis mellifera*	Antioxidant, antihyperglycemic, and antidiabetic, restoration of reproductive functions, and reduction of endometrial hyperplasia and membrane inflammation	[63, 64]
	Barley, royal jelly	Decrease in free radicals and increase in antioxidant enzymes	[65, 66]
	Cinnamon, *Ficus asperifolia* water extract	Reduction of oxidative stress and control of lipid components, improving fertility	[67, 68]

Negative	*Olea europaea* water-alcohol extract and permethrin	Decline in reproductive function even at doses as low as 50 and 35 mg/kg, respectively	[69, 70]
	*Tripterygium* glycoside extract	Inhibition of the activity of hormone receptors critical in the fertilization process	[71]
	The dose of *Ricinus communis* L. water extract was higher than 100 mg/ml, and the dose of ethanol extract from *Spondias mombin* L. and red cultivar *Allium cepa* methanol extract exceeded 100 mg/kg	Adverse fertility	[72–74]

## Data Availability

No data were used to support this study.
